# Poster Session II - A244 URINARY METABOLITES IDENTIFY FIRST-DEGREE RELATIVES AT RISK OF DEVELOPING CROHN’S DISEASE

**DOI:** 10.1093/jcag/gwaf042.244

**Published:** 2026-02-13

**Authors:** T Sharar Fischler, F A Guevara Agudelo, S Jeong, R Chen, A Griffiths, C Bernstein, H Steinhart, K Jacobson, S Lee, K Croitoru, W Turpin

**Affiliations:** University of Toronto Temerty Faculty of Medicine, Toronto, ON, Canada; University of Toronto Temerty Faculty of Medicine, Toronto, ON, Canada; University of Toronto Temerty Faculty of Medicine, Toronto, ON, Canada; University of Toronto Temerty Faculty of Medicine, Toronto, ON, Canada; The Hospital for Sick Children, Toronto, ON, Canada; University of Manitoba Max Rady College of Medicine, Winnipeg, MB, Canada; University of Toronto Temerty Faculty of Medicine, Toronto, ON, Canada; BC Children’s Hospital, Vancouver, BC, Canada; University of Toronto Temerty Faculty of Medicine, Toronto, ON, Canada; University of Toronto Temerty Faculty of Medicine, Toronto, ON, Canada; University of Toronto Temerty Faculty of Medicine, Toronto, ON, Canada

## Abstract

**Background:**

Early diagnosis is essential for optimal management and prognosis in Crohn’s disease (CD). Identifying individuals at risk before the onset of clinical symptoms through non-invasive biomarkers remains a challenge. Urinary metabolomic assessment may provide such a tool, as it can capture both systemic and gut-derived metabolic changes.

**Aims:**

Investigate whether baseline urinary metabolomic profiles can identify individuals at risk of developing CD.

**Methods:**

We analyzed urine metabolomics from the CCC-GEM Project, a prospective cohort of healthy first-degree relatives (FDRs) of CD patients. A nested case-control design compared FDRs who later developed CD (pre-CD) with matched control FDRs who remained disease-free. Untargeted urine metabolomics was performed using Metabolon untargeted panel. Conditional logistic regression assessed urine metabolite associations with future CD development, adjusting for matched sets by age, sex, geography, and follow-up duration. Correlations between significant metabolites and fecal calprotectin (FCP), C-reactive protein (CRP), and the urinary fractional excretion ratio of lactulose to mannitol (LMR) were evaluated using Spearman’s rank correlation. False discovery rate correction was applied (q < 0.05).

**Results:**

Among 302 participants (64 pre-CD, 238 controls), we identified 21 urinary metabolites significantly associated with subsequent CD development (q = 0.019–0.047). These included microbiota-derived products of aromatic amino acid fermentation (e.g., phenol sulfate, phenylacetyl conjugates) and metabolites from host and microbial tryptophan catabolism (e.g., indoxyl, quinolinate derivatives). Fifteen of these significant metabolites correlated with CRP, nearly all positively. FCP correlated only with 3,5-dihydroxyphenylpropionate (ρ=–0.19, q = 0.03), while no significant correlations were observed with LMR.

**Conclusions:**

In this large prospective cohort of healthy FDRs, we identified urinary metabolomic signatures that predict the future onset of CD. Urine-based metabolomics may offer a promising, non-invasive approach to identify individuals at high risk of developing CD, with potential applications in risk stratification and prevention strategies.

**Funding Agencies:**

CCC, CIHRHelmsley Charitable trust

